# Targeting of CRMP-2 to the Primary Cilium Is Modulated by GSK-3β

**DOI:** 10.1371/journal.pone.0048773

**Published:** 2012-11-21

**Authors:** Young Ou, Ying Zhang, Min Cheng, Jerome B. Rattner, Ina Dobrinski, Frans A. van der Hoorn

**Affiliations:** 1 Department of Biochemistry and Molecular Biology, University of Calgary, Calgary, Canada; 2 Department of Cell Biology and Anatomy, Faculty of Medicine, University of Calgary, Calgary, Canada; 3 Department of Comparative Biology & Experimental Medicine, Faculty of Veterinary medicine, University of Calgary, Calgary, Canada; King's College London, United Kingdom

## Abstract

CRMP-2 plays a pivotal role in promoting axon formation, neurite outgrowth and elongation in neuronal cells. CRMP-2′s role in other cells is unknown. Our preliminary results showed CRMP-2 expression in cilia of fibroblasts. To localize CRMP-2, define its role and study the regulation of CRMP-2′s expression in cilia we carried out the following experiments. We find that in fibroblasts CRMP-2 localizes to the centrosome and is associated with the basal body and -at a low level- is present in primary cilia. Phosphorylated pCRMP-2 can only be detected at the basal body. RNAi knockdown of CRMP-2 interfered with primary cilium assembly demonstrating a critical requirement for CRMP-2. Deletion analysis of CRMP-2 identified a 51 amino acid sequence in the C-terminus that is required for targeting to the basal body and primary cilium. This domain contains GSK-3β phosphorylation sites as well as two repeats of the VxPx motif, previously identified as a cilium targeting signal in other primary cilium proteins. To our surprise, mutation of the CRMP-2 VxPx motifs did not eliminate primary cilium targeting. Instead, mutation of the GSK-3β phosphorylation sites abolished CRMP-2 targeting to the primary cilium without affecting basal body localization. Treatment of cells with lithium, a potent GSK-3β inhibitor, or with two specific GSK-3β inhibitors (the L803-mts peptide inhibitor and CHIR99021) resulted in cilium elongation and decreased basal body levels of pCRMP-2 as well as increased levels of total CRMP-2 at the primary cilium. In summary, we identified CRMP-2 as a protein critically involved in primary cilia formation. To our knowledge this is the first demonstration of modulation of primary cilium targeting by GSK-3β.

## Introduction

The primary cilium is an antenna-like structure, protruding from the surface of many different types of mammalian cells. Many signalling proteins, including somatostatin receptor 3, platelet-derived growth factor receptor alpha, polycystins and Patched, are concentrated at the ciliary membrane [1]–[6]. In addition, some extracellular matrix receptors are also localized to primary cilia [7], [8]. Accumulation of these signaling molecules at the primary cilium allows it to serve as a sensory organelle, detecting molecular and mechanical changes in the extracellular environment and relaying these changes to the cell body where they induce a variety of cellular responses [9]. Primary cilia are also essential during embryonic development for correct functioning of the vertebrate hedgehog signaling pathway [4], [10]–[12]. Abnormal primary cilia appear involved in obesity, polycystic kidney disease and cancer, as well as a number of other diseases [for reviews, see [13]–[16]]. In cycling cells, primary cilium expression is coupled to the cell cycle [17]. The ciliary axoneme first forms in G1 phase. With cell cycle progression, the primary cilium is resorbed, and the basal body from which it arose reverts to a centrosome and subsequently participates in the establishment of the spindle poles [18]. After cell division, the centrosome once again acts a basal body, producing the primary cilium. Structurally, a mammalian primary cilium consists of a centriole (basal body) and a membrane-bound ciliary axoneme, which is comprised of nine doublet microtubule bundles. Like polarized microtubule-based structures such as axon and dendrites, the microtubule-based primary cilium has polarity, with the minus end associated with the basal body and the plus end pointed away from the cell body. In neurons, many proteins specifying axon polarity have been identified, including collapsing response mediator protein 2 (CRMP2), a cytosolic phosphoprotein enriched in the axon shaft and growth cone [19]. CRMP-2 is a GSK-3β substrate and a microtubule-binding protein. GSK-3β regulation of CRMP-2 phosphorylation affects neurite outgrowth and elongation [20]–[23]. However, the location and function of CRMP-2 remains unknown in non-neuronal cells, although a recent study suggested that in these cells CRMP-2 is involved in endocytosis [24]. In a previous study, we showed that GSK-3β activity can be inhibited by lithium, causing elongation of primary cilia [25]. In this study, we characterized the GSK-3β substrate CRMP-2 in human foreskin fibroblasts, which have retained the ability to express primary cilia. We found that CRMP-2 localizes to the centrosome. During periods of primary cilium formation, CRMP-2 associates with the basal body and is also present at low levels at the primary cilium. We demonstrate that CRMP-2 is required for cilium biogenesis and that the CRMP-2 protein sequence contains a novel cilium targeting motif composed of GSK-3βsites. Dephosphorylation of the CRMP-2 GSK-3β sites is required for primary cilium localization.

**Table 1:** CRMP-2 Mutants.

CRMP-2 mutant	aa	primer sequence (5′–3′)
Mut1-GFP	1–389^1^	5′- GCT TCG AAT TCC GCC ACC ATG TCT TAT CAG GGG AAG a-3′ (forward) and 5′- TAC CG TCG ACC GGC TGC ATT GGT GCT GGT CAC-3′ (reverse)
Mut2-GFP	1–440^1^	5′- GCT TCG AAT TCC GCC ACC ATG TCT TAT CAG GGG AAG A-3′ (forward) and 5′- CGA CCG TCG ACC CGC GGC ACT CCA TGC CTT C-3′ (reverse)
Mut3-GFP	441–572^1^	5′- GCT TAG AAT TCC GCC ACC ATG GGC TCC CCA CTG GTG GTC ATC-3′ (forward) and 5′- TAC CGT CGA CTG GCC CAG GCT GGT GAT GTT G -3′ (reverse)
Mut4-GFP	441–560^1^	5′- GCT TAG AAT TCC GCC ACC ATG GGC TCC CCA CTG GTG GTC ATC-3′ (forward) and 5′-GAC CGT CGA CGC CAC GAT ACG CTG GGT GGT-3′ (reverse)
Mut5-GFP	441–547^1^	5’- GCT TAG AAT TCC GCC ACC ATG GGC TCC CCA CTG GTG GTC ATC-3′ (forward) and 5′-GAC CGT CGA CT CAT CAA TCT GAG CAC CAG A-3′ (reverse)
Mut6-GFP	441–535^1^	5′- GCT TAG AAT TCC GCC ACC ATG GGC TCC CCA CTG GTG GTC ATC-3′ (forward) and 5′-ATT AGT CGA CTG GTG CAG GTT CCG GAC AGG-3′ (reverse)
Mut7-GFP	484–535^1^	5′-GCT TAG AAT TCC GCC ACC ATG GCA AGG AGC AGG CTG GCT G-3′ (forward) and 5′-ATT AGT CGA CTG GTG CAG GTT CCG GAC AGG-3′ (reverse)
Mut8-GFP	Δ484–535^2^	5′-CCG GAA TTC GAT ATC CAC CAG TCT GGA TTC AGT TTG-3′ (forward) and 5′- GAA CGG GAT ATC TGC CTT GAT ACG CTT GTA AAC-3′ (reverse)
Mut9-GFP	ΔVxPx^3^	5′- GCT TCG AAT TCC GCC ACC ATG GCG ACG GCC AAG ACA GCC ACT GCA GCC TCC TCG GCC AGG ACG TCT CCT GCC AAG CAG CAG GCC CCA CCT GTC CGG AAC CTG GAC CAG TCG ACT CCA-3′ (forward) and 5′-ATT AGT CGA CTG GTG CAG GTT CCG GAC AGG-3′ (reverse)
Mut10-GFP	ΔGSK^4^	5′- GCT TCG G AAT TCC GCC ACC ATG GTG GCG CCC AAG GCA GTC GCT CCA GCC GCC GCG GCC AGG ACG TCT CCT GCC AAG CAG CAG GCC CCA CCT GTC CGG AAC CTG CAC CAG TCG ACT CCA-3′ and 5′-ATT AGT CGA CTG GTG CAG GTT CCG GAC AGG-3′ (reverse)

Notes:

1) indicated are the CRMP-2 amino acid residues retained in the indicated mutant.

2) indicated are the CRMP-2 amino acid residues deleted in the indicated mutant.

3) Mut9-GFP contains mutated VxPx motifs.

4) Mut10-GFP contains mutated GSK-3β phosphorylation sites.

## Materials and Methods

### Cell culture

Human foreskin fibroblasts [25] and human retinal pigmented epithelium RPE cells (ATCC CRL-4000) were grown in DMEM (Gibco/BRL) supplemented with 10% fetal calf serum. All work was carried out in accordance with Biohazardous Materials approval, University of Calgary. H293 fibroblasts were only used in the immunoprecipitation experiments. For preparation of log phase cells, cells were collected by trypsin/EDTA (Gibco/BRL) treatment and re-grown overnight on coverslips in fresh medium. For preparation of quiescent cells, log phase cells were incubated in a medium lacking serum for 48 hrs. For lithium drug treatment, cells were serum-starved for two days prior to treatment, and the concentration and duration of lithium treatment were as described previously [25] and in the text. For GSK-3β inhibition experiments cells were treated with the cell-permeable, specific GSK-3β peptide inhibitor L803-mts [26], [27] (EMD) or the specific GSK-3β inhibitor CHIR-99021 (EMD). Prior to treatment, cells were serum-starved for two days, and concentrations and duration of treatments are indicated in the text.

**Figure 1:**
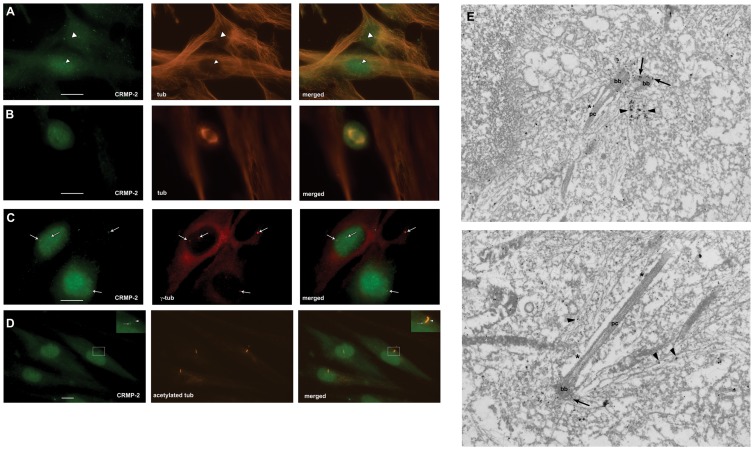
CRMP-2 localizes to the centrosome/basal body. Human foreskin fibroblasts were stained with anti-CRMP-2 antibody (green signal), and co-stained with anti-β-tubulin antibody (A and B; red signal) to illustrate microtubule organizing center and spindle poles, respectively, anti-γ-tubulin antibody (red signal) to reveal the centrosomes (C) or anti-acetylated tubulin antibody (red signal) to visualize the primary cilia (D). A) CRMP-2 is at the centrosome in interphase cells (arrowheads). B) CRMP-2 shows diffuse staining in mitotic cells. C) CRMP-2 and γ-tubulin colocalize (arrows). D) Small amounts of CRMP-2 are at the basal body (arrow) and primary cilia (arrowhead): the inset is an enlarged image of the indicated region. The last column in panels A–D shows merged images. Bars indicate 10 μm. Magnification: 100X. E) Immuno electron microscopy shows CRMP-2 at the basal body (bb: arrows) and the primary cilium (pc). Arrowheads indicate CRMP-2 at microtubules emanating from the basal body, and asterisks indicate CRMP-2 occasionally observed at the shaft of the primary cilium. Magnification: 50,000X.

### Antibodies

Anti-acetylated α- tubulin, anti-β-tubulin, and anti-γ-tubulin antibodies were purchased from Sigma (St Louis, MO). Antibodies against GSK-3β, phospho (Ser-9) GSK-3β and CRMP-2 were purchased from Cell Signaling Technology (Boston, MA). Anti-phospho (T514) CRMP-2 antibody was purchased from Abcam (Cambridge, MA). HRP-conjugated secondary antibody and Cy^3^-, and Alex^488^-labeled secondary antibodies were purchased from Jackson ImmunoResearch (West Grove, PA) and Molecular Probes (Eugene, OR), respectively.

**Figure 2:**
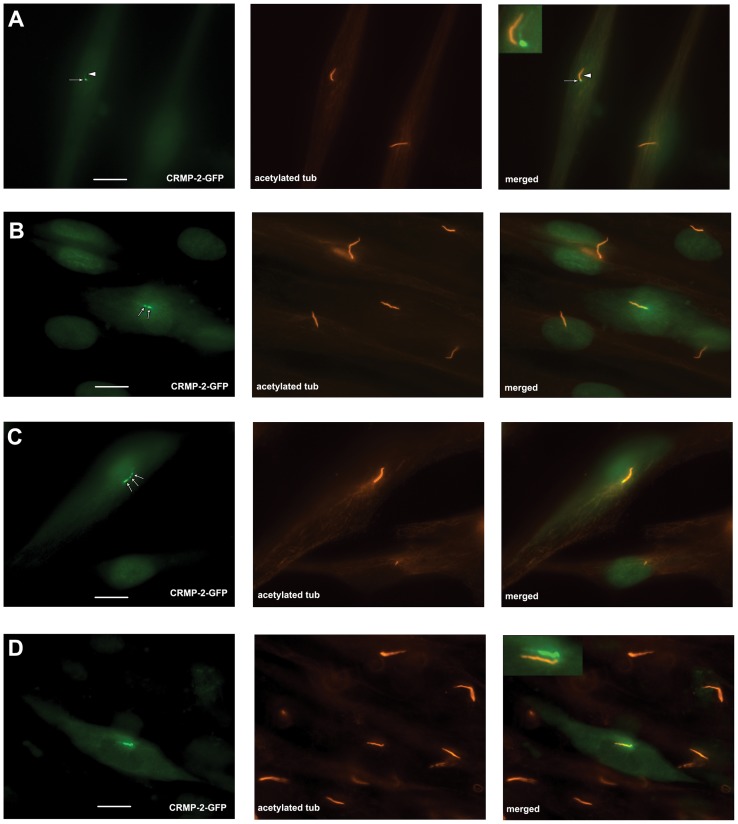
CRMP-2-GFP expression pattern in fibroblasts. Human foreskin fibroblasts were transfected with CRMP-2-GFP (green signal), fixed and stained with anti-acetylated tubulin antibodies (red signal). The last column shows merged images. A) GFP signal was observed at the basal body (arrow) and –at a low level- at the primary cilium (arrowhead). The inset shows an offset of an enlarged part of the image to compare staining patterns for acetylated tubulin and CRMP-2-GFP. B) and C) show examples of transfected cells with CRMP-2-GFP in concentrations in the primary cilium (arrows). D) Shows an example of the unequal distribution of CRMP-2-GFP occasionally observed: the inset shows an offset for comparison of the signals. Bars indicate 10 μm. Magnification: 100X.

### RNA isolation and RT-PCR

RNA was isolated from human foreskin fibroblasts using TRIzol (Invitrogen) as recommended by the manufacturer (Invitrogen). RT-PCR was performed as described [25] **.** Briefly, RNA was transcribed into cDNA using M-MLV (Invitrogen). For each sample, 100 ng of random primers was used, and a total volume of 20 μl of cDNA was generated, 2.5 μl of which were used as template for PCR in a 50 μl reaction volume. For each primer set, 30 cycles of PCR were carried out under the following conditions: a denaturing step for 30 s at 94°C, an annealing step for 45 s at 60°C, and an elongation step for 1.5 min at 72°C. For PCR amplification of full length CRMP-2 (572 amino acid residues), the forward primer was 5′- GCT TCG AAT TCC GCC ACC ATG TCT TAT CAG GGG AAG A -3′, and the reverse primer was 5′- TAC CGT CGA CTG GCC CAG GCT GGT GAT GTT G -3′. PCR fragments were analyzed in a 1.2% agarose gel, and sequence analysis of the cDNA fragments was determined at the University of Calgary Core DNA Services.

**Figure 3:**
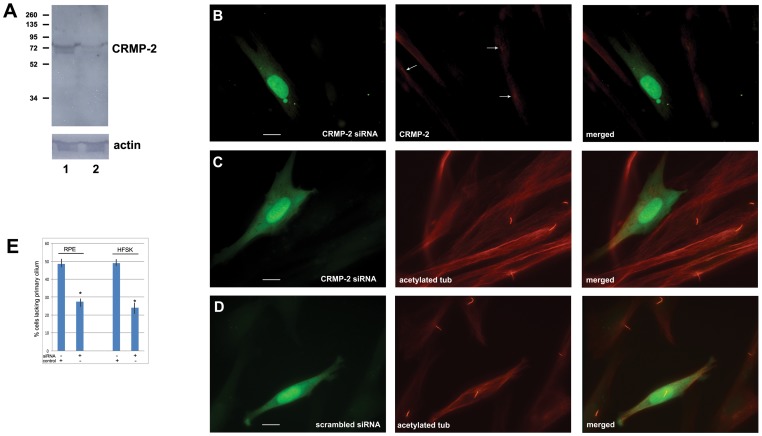
CRMP-2 is required for ciliogenesis. CRMP-2 expression was knocked down using siRNA. Efficacy was measured in human foreskin fibroblasts by western blotting (A) and by immunofluorescence of transfected cells (B). A) Human foreskin fibroblasts were transfected with iLenti-Scramble-siRNA-GFP (lane 1) or iLenti-CRMP-2-siRNA-GFP (lane 2) and endogenous CRMP-2 protein expression was analyzed by western blotting using anti-CRMP-2 antibodies. Both constructs express GFP independent of the siRNA, as a marker for cells positively transfected. Blots were reprobed using anti-actin antibodies. B) Human foreskin fibroblasts were transfected with the constructs listed in A) and analyzed for CRMP-2 protein expression in GFP positive cells. Cells expressing CRMP-2 siRNA (green signal) have a significantly reduced level of CRMP-2 (red signal); GFP-negative cells show normal CRMP-2 (arrows). C) Primary cilia in human foreskin fibroblasts transfected with CRMP-2 siRNA (green signal) were visualized using anti-acetylated tubulin antibodies (red signal). A majority of CRMP-2 siRNA expressing cells lacked a primary cilium. Surrounding untransfected cells were not affected. D) A scrambled siRNA (green signal) had no effect on cilium formation using anti-acetylated tubulin antibodies (red signal). In B0, C) and D) bars indicate 10 μm. Magnification: 100X. E) Quantitation of CRMP-2 siRNA transfection experiments in RPE cells and human foreskin fibroblasts (HFSK) shows a significant difference (P<0.05) between the percentage of cells lacking a primary cilium after CRMP-2 siRNA expression (siRNA) compared to cells expressing scrambled siRNA (control). Multiple experiments were done and >200 GFP-positive cells were measured in each condition.

### Plasmid constructions and DNA transfection

All CRMP-2-GFP plasmids (wild type and mutants) were made using PCR amplification of the above-mentioned RT-PCR products, followed by digestion with restriction enzymes Eco RI and Sal I, and insertion into the appropriate sites of the pMAXGFP vector (Lonza). Primers used for construction of CRMP-2 mutants are listed in Table 1. For HA-Mut7, the DNA fragment was amplified using the forward primer 5′ - GCT TAC GAA TTC CGC CAC GCA AGG AGC AGG CTG GCT G and the reverse primer 5′-att agt cga ctg gtg cag gtt ccg gac agg-3′. After restriction digestion with Eco RI and Sal I, the fragment was inserted into the corresponding sites of the pCI vector (Promega) containing an HA tag. HA-KLC-3 and GFP-KLC-3 were described previously [28]. Plasmid constructs were transfected into log phase cells using Extremegene under conditions recommended by the manufacturer (Roche).

**Figure 4:**
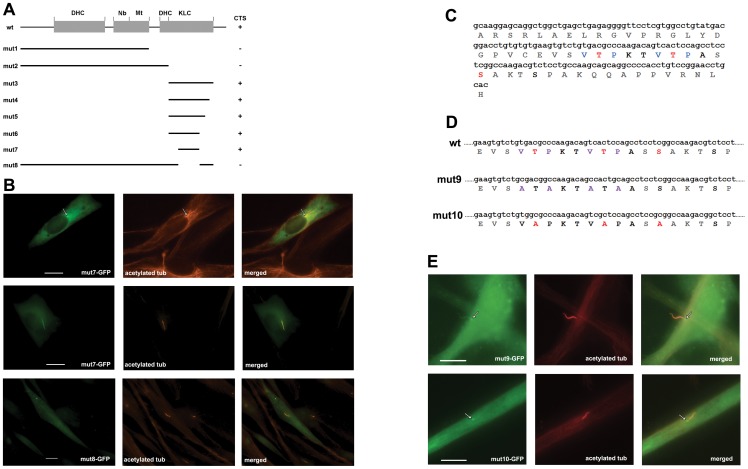
CRMP-2 mutation analysis delineates the primary cilium targeting sequence. A) Domain structure of wild type CRMP-2 (wt) and corresponding regions present in the mutants (mut1 - mut8) are indicated. DHC, dynein heavy chain interacting domain; Nb, Numb interacting domain; Mt, microtubule-binding domain; KLC, kinesin light chain interacting domain. CTS, cilium targeting capability, based on observations of mutant protein expression patterns in transfected fibroblasts. B) Immunofluorescence analysis of human foreskin fibroblasts expressing mut7-GFP or mut8-GFP as indicated. Human foreskin fibroblasts were transfected with mut7-GFP or mut8-GFP (green signal) and analyzed using anti-acetylated tubulin antibodies (red signal). Mut7-GFP localizes to the basal body (top panels: arrow) in cells lacking a primary cilium, and to the cilium in cells that express the organelle (middle panels). Mut8-GFP does not localize to basal body or cilium. Bars indicate 10 μm. Magnification: 100X. C) Nucleotide sequence and deduced amino acid sequence of the CRMP-2 51 residue C-terminal region present in mut7. Val and Pro residues in the VxPx sequences are indicated in blue and Thr and Ser residues in known GSK-3β phosphorylation sites are indicated in red (T509, T514 and S518). D) Point mutations were introduced to mutate the VxPx sites to AxAx (mut9: purple lettering) and GSK-3β phosphorylation sites to Ala (mut-10: red lettering). E) Analysis of the localization of mut9-GFP (top panels) and mut10-GFP (bottom panels) (green signals) was done by transfection of human foreskin fibroblasts and staining for acetylated tubulin (red signals). Although the intensity of mut9-GFP was lower than wild type, mut9-GFP was observed to localize to both the basal body (arrow) and the cilium. The bottom panels show that mut10-GFP only localizes to the basal body (arrow), not the cilium. Bars indicate 10 μm. Magnification: 100X.

### siRNA experiments and primary cilium growth assay

A set of lentiviral vector-based siRNA plasmids (pILenti-siRNA-GFP) was purchased from Applied Biological Materials Inc (Vancouver, Canada). This set of plasmids expresses shRNA duplexes that target the following CRMP-2 sequences: AGCGATCGTCTTCTGATCAAAGGAGGTAA, AAGATGGGTTGATCAAGCAAA, and ACGGATTGCCAGATTTATGAAGTACTGAG. For control, the same vector plasmid expressing a scrambled shRNA was used. Plasmids were transfected into cells using Extremegene under conditions recommended by the manufacturer (Roche). After transfection, cells were cultivated for 72 hrs in complete culture medium and for an additional 48 hrs in serum-free medium. Cells were then either fixed with 2% paraformaldehyde for immunofluorescence microscopy or collected for analysis by semi-quantitative RT-PCR.

**Figure 5:**
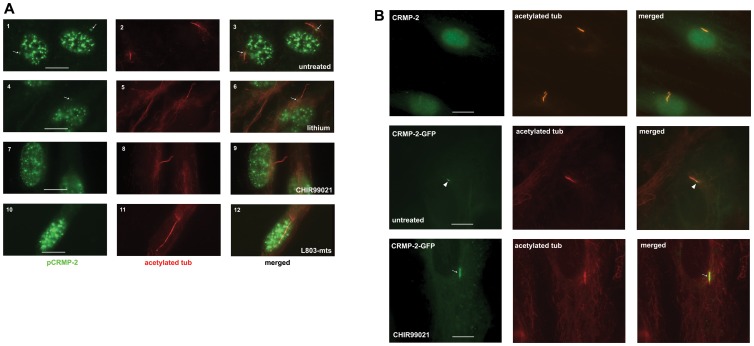
Inhibition of GSK-3β enhances CRMP-2 levels in elongated primary cilia. A) The effect of inhibition of GSK-3β on amount and localization of endogenous phosphorylated pCRMP-2 was analyzed by indirect immunofluorescence using anti-pCRMP-2(Thr514) antibodies (green signals) in untreated RPE cells and in RPE cells treated with the indicated GSK-3β inhibitors: lithium, CHIR99021 and the L803-mts peptide inhibitor. Primary cilia were visualized using anti-acetylated tubulin antibodies (red signals). Arrows point to pCRMP-2 at the basal body. No phosphorylated pCRMP-2 was seen in primary cilia using this method. The right column shows merged images. Bars indicate 10 μm. Magnification: 100X. B) To analyze the effect of inhibition of GSK-3β on total CRMP-2 protein, we attempted to analyze endogenous CRMP-2 in RPE cells before and after treatment with the GSK-3β inhibitor CHIR99021 (top panels). However, signal was too low to provide for reliable quantitative data and we therefore resorted next to using RPE cells transfected with CRMP-2-GFP. CRMP-2-GFP in transfected RPE cells was analyzed in untreated cells (“untreated”, middle panels) or after treatment with CHIR99021 (“CHIR99021”, bottom panels). Primary cilia were visualized using anti-acetylated tubulin antibodies (“acetylated tub”, red signals). Note the presence of CRMP-2-GFP in untreated cells predominantly at the basal body (middle panels, arrowhead). GSK-3β inhibition caused a readily detectable increase in CRMP-2-GFP in primary cilia (bottom panels, arrow). Bars indicate 10 μm.

### Immunoprecipitations

H293 cells were co-transfected with CRMP-2 Mut7 and HA-KLC3 or HA-CRMP-2 Mut7 and GFP-KLC3. After transfection, cells were incubated for 24 hrs to allow protein expression, and cell extracts were prepared in lysis buffer (10 mM Tris pH 7.4, 150 mM NaCl, 10 mM KCl, 1 mM EDTA pH 8.0, 0.5% DOC, 0.5% Tween-20, 0.5% NP-40, 1x proteinase inhibitor cocktail (Roche)). Protein extracts were pre-cleared by mixing with 20 μl protein G Sepharose beads and incubated for overnight at 4°C with either anti-HA antibody or anti-GFP antibody as stated in the text. Following incubation, the beads were precipitated and washed three times with cell lysis buffer. After a final wash, proteins were eluted from the beads with SDS-sample buffer, and analyzed by gel electrophoresis and western blot.

### Gel electrophoresis and western blot

Gel electrophoresis and western blot analysis were carried out as described previously [29]. In short, proteins were boiled in loading buffer, separated on 10% acrylamide SDS-PAGE gels, electrophoretically transferred onto polyvinylidene fluoride membranes (Amersham Biosciences), blocked overnight at 4 °C in blocking buffer (54 mM Tris, pH 7.5, 150 mM NaCl, 0.05% Nonidet P-40, 0.05% Tween 20, 5% dry nonfat milk), and analyzed using primary antibodies followed by HRP-conjugated secondary antibody. Prestained Protein Ladder SM0671 (Fermentas) was used as a size marker. LumiGLO substrate (Kirkegaard & Perry Laboratories, Inc.) was used to develop the blot. The luminescent image was captured using a 3000 Versa-Doc Imaging System (Bio-Rad).

### Immunofluorescence microscopy

Indirect immunofluorescence microscopy (IIF) was performed as described previously [30], [31]. Briefly, cells were fixed either in cold methanol for anti-CRMP-2 antibody staining or in 2% paraformaldehyde in PBS for direct visualization of GFP-expressing cells. The cells then were stained with primary antibodies, followed by secondary antibodies at a concentration recommended by the manufacturers. Images were obtained using a Zeiss microscopy Axiovert 200M equipped with a CCD camera and controlled with AxioVision 4.8 software. The intensity of CRMP-2 signal at the basal body before and after treatment of cells with GSK-3β inhibitors was measured and quantitated as described by Mahjoub and Stearns [32]. For statistical analysis of quantitated immunofluorescence data we used the Student's t-test and the chi-square test.

### Immuno-electron microscopy

Cells were fixed in cold methanol and stained with anti-CRMP-2, followed by incubation with a secondary antibody conjugated with Nano-gold particles (Nanoprobes). Nanogold was silver enhanced with HQ Silver (Nanoprobes) and cells were further processed by fixation in 2% glutaraldehyde and 1% osmium tetroxide. Cells were then counter-stained with 2% uranyl acetate, dehydrated in ethanol, embedded in Epon resin, and sectioned as described previously [25]. The samples were examined using a Hitachi-7650 microscope [33].

## Results

### CRMP-2 is located at the centrosome in human foreskin fibroblasts

We first confirmed CRMP-2 RNA expression in human foreskin fibroblasts. Western blot analysis confirmed CRMP-2 protein expression in human foreskin fibroblasts with the characteristic doublet pattern seen in neuronal cells (Fig. S1), the slower migrating band representing the phosphorylated form, while the other represents the non-phosphorylated form [21], [34].

Immunofluorescence microscopy using anti-CRMP-2 and anti-β-tubulin antibodies revealed that CRMP-2 is present at the centrosome during interphase (Fig. 1A, arrowheads) in all cells examined (Fig. 1C, arrows indicate centrosomes stained for CRMP-2 and γ-tubulin). CRMP-2 was not detected at the mitotic spindle pole (Fig. 1B).

### CRMP-2 associates with the basal body

The association of CRMP-2 with the interphase centrosome suggested a possible role for CRMP-2 in centrosome function. We first analyzed where CRMP-2 protein is located in fibroblasts that express a primary cilium and found that in serum-starved cells CRMP-2 co-localizes with the basal body in all cells examined (Fig. 1D, arrow). In approximately 5% of the cells CRMP-2 was also present –albeit at a low level- at the primary cilium (Fig. 1D, arrowhead, inset shows an example). To confirm this we carried out immuno-electron microscopy using anti-CRMP-2 antibodies and show that CRMP-2 is associated with the basal body and microtubules emanating from it (Fig. 1E, upper panel: arrows and arrowheads, respectively). Occasionally CRMP-2 was observed at the primary cilium shaft (Fig. 1E, asterisk).

### CRMP-2-GFP expression pattern at the primary cilium

To investigate the mechanism underlying the localization of CRMP-2 to basal body and primary cilium, we expressed a CRMP-2-GFP fusion protein in fibroblasts. Expression of the CRMP-2-GFP fusion protein was confirmed by western blot analysis. We observed that the CRMP-2-GFP fusion protein localized predominantly to the basal body (Fig. 2A, arrow). Small amounts were present throughout the primary cilium (Fig. 2A, arrowhead; inset shows offset of the two signals to visualize both signals in the primary cilium) indicating that the localization of CRMP-2-GFP and endogenous CRMP-2 are similar.

We noted that in approximately 16% of CRMP-2-GFP transfected cells the fusion protein was present in concentrations along the shaft of the primary cilium (Fig. 2B, Fig. 2C, arrows) in addition to its presence at the basal body and in some cells fusion protein started at the basal body and extended inside the primary cilium (Fig. 2D). The location of the concentrations varied from cell to cell.

### CRMP-2 is required for cilium assembly

To investigate a possible function of CRMP-2, we performed RNAi assays to knock down the level of CRMP-2. We used a lentiviral-based vector that expresses GFP and CRMP-2 shRNA to allow identification of transfected cells vs untransfected cells. RPE cells and human foreskin fibroblasts (HFSK) were used in these experiments and those with GFP expression were used for quantitative analysis. Western analysis of cells transfected with CRMP-2-siRNA-GFP DNA show reduced levels of CRMP-2 in comparison to cells transfected with scrambled siRNA-GFP control DNA (Fig. 3A). CRMP-2 is reduced in CRMP-2-siRNA-GFP expressing cells (Fig. 3B), but not in untransfected cells (Fig. 3B, arrows) or cells expressing a scrambled siRNA (Fig. S2). To observe an effect of reduced CRMP-2 on primary cilia we stained primary cilia in transfected cells with anti-acetylated tubulin antibody. This showed that cells transfected with CRMP-2 siRNA-GFP lacked a primary cilium (Fig. 3C), whereas cells that express scrambled siRNA-GFP express a primary cilium (Fig. 3D). In untransfected cells in the same experiment, primary cilium formation was unaffected (Fig. 3C and 3D). Quantitation of these results (Fig. 3E) showed that 48.5% and 49% of RPE cells and HFSK fibroblasts transfected with CRMP-2 siRNA lacked a primary cilium (Fig. 3E). In contrast, 27% and 24% of RPE cells and HFSK fibroblasts transfected with a scrambled siRNA lacked a primary cilium. The decrease in primary cilia was significant (P<0.05) and indicated that CRMP-2 is important for primary cilium biogenesis in these cell lines.

### Basal body and cilium targeting signals are located in the CRMP-2 C-terminus

The CRMP-2 protein sequence contains putative protein interaction motifs for cytoplasmic dynein heavy chain, Numb, microtubules and kinesin light chain (Fig. 4A) [35]–[38]. To determine a role in targeting of CRMP-2 to the basal body and/or primary cilium, we analyzed deletion mutants linked to GFP that retained one or more motifs (Fig. 4A). Western blotting was used to confirm expression of the fusion proteins. Transfection showed that mutants mut1 and mut2, which lacked the C-terminus, did not localize to the basal body or primary cilium. In contrast, mutants mut3, mut4, mut5, mut6 and mut7 that retain parts of the kinesin light chain-binding domain were present at the basal body in cells that lacked a primary cilium (Fig. 4B, upper panels; arrow shows the results for mut7, which are identical to those for mut3 – mut6) and at both the basal body and the primary cilium in cells expressing a primary cilium (Fig. 4B, middle panels). This indicated that the cilium targeting signal (CTS) of CRMP-2 is in a 51 amino acid sequence (amino acid residues 484 – 535) shown in Fig. 4C. Western blotting analysis of the 51 amino acid sequence linked to GFP showed two bands (Fig. S3) suggesting that -similar to full-length CRMP-2 [21], [34]- it may also be subject to modification by phosphorylation. Further deletion of the 51 amino acid sequence from either the N- or C-terminus resulted in loss of cilium targeting. Deletion of the 51 amino acid sequence from CRMP-2 (Fig. 4A, mut8) prevented targeting to basal body or primary cilium (Fig. 4B, bottom panels).

### GSK-3β phosphorylation sites are required for targeting of CRMP-2 to the primary cilium

Analysis of the 51-amino acid sequence identified putative motifs: two VxPx repeats (Fig. 4C, blue lettering) and three GSK-3β phosphorylation sites (T509, T514, and S518; Fig. 4C, red lettering). VxPx and the related motif RVxPx and KVHPSST are involved in cilium targeting of rhodopsin, polycystin-2 and polycystin, respectively [39]–[42]. To analyze a role for CRMP-2 VxPx in cilium targeting we made point mutations changing VTPK to ATAK and VTPA to ATAA (Fig. 4D, mut9). Transfection assays revealed that mut9-GFP fusion protein was present at both the basal body and primary cilium in 35% of transfected cells (n = 37) (Fig. 4E, top panels; arrow points to basal body). Quantitation of the mut9-GFP signal intensity in comparison to mut7-GFP showed that it was reduced by 45% of mut7-GFP (P<0.05). These results indicate that the CRMP-2 VxPx sequences are not absolutely required for targeting CRMP-2 to the primary cilium or basal body.

**Figure 6:**
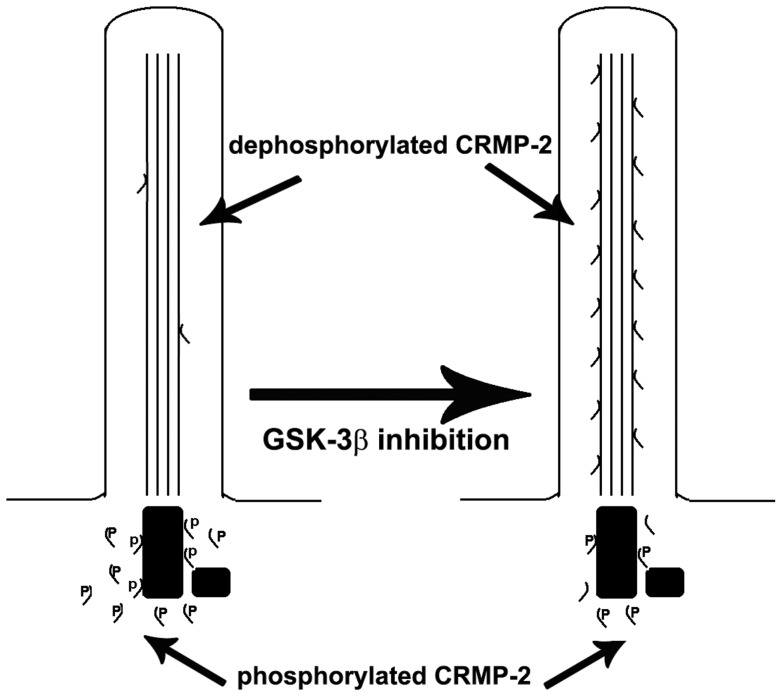
Model for regulation of CRMP-2 localization to primary cilia. The model schematically presents that the majority of CRMP-2 in normal serum-starved cells is phosphorylated by GSK-3β and resides at the basal body. After GSK-3β inhibition or mutation of CRMP-2 GSK-3β phosphorylation sites, CRMP-2′s presence in the primary cilium is enhanced.

We next prepared point mutations in all three GSK-3β phosphorylation sites (T509, T514, and S518 to alanine) (Fig. 4D, mut10). Transfection experiments showed mut10-GFP fusion protein was present only at the basal body, not in the primary cilium (n = 63) (Fig. 4E, bottom panels, arrow). This result strongly suggested that GSK-3β is involved in targeting CRMP-2 to the primary cilium.

### GSK-3β regulates CRMP-2 targeting to the primary cilium

CRMP-2 is a physiological substrate of GSK-3β [21], [43], [44]. To explore a role for GSK-3β in CRMP-2 localization to primary cilia, we exploited our earlier observation that lithium, an inhibitor of GSK-3β, induces primary cilium elongation [25]. Treatment of foreskin fibroblasts with lithium inhibited phosphorylation of CRMP-2 (Fig. S4), similar to its effect on other GSK-3β substrates Map1b and β-catenin [25]. This observation confirmed that a majority of CRMP-2 is in a phosphorylated state in normal (untreated) serum-depleted cells [21]. We next examined the effect of GSK-3β inhibition on the localization of endogenous CRMP-2. We employed three different inhibitors; Li, the GSK-3β inhibitor peptide L803-mts [26], [27], [45], [46] and CHIR99021 (see Materials and Methods). We first measured the length of cilia in treated and untreated cells. In untreated cells we observed an average cilium length of 4.65 μm (n = 157; SD = 1.10 μm). In cells treated with Li, CHIR99021 and the L803-mts peptide inhibitor the average cilium length was 8.99 μm (n = 67; SD = 2.17 μm), 5.61 μm (n = 123; SD = 1.62 μm) and 9.99 μm (n = 58; SD = 4.12 μm), respectively. The effects of Li and peptide inhibitor L803-mts on cilium length were significant (P<0.05), whereas the effect of CHIR99021 was measurable but modest.

Next we analyzed the effect of the three GSK-3β inhibitors on the localization of phosphorylated CRMP-2, because our above results had suggested that inhibition of GSK-3β results in a reduction of the phosphorylation level of CRMP-2 and its subsequent translocation from the basal body to the cilium. To monitor and quantitate phosphorylated pCRMP-2 we employed the specific anti-pCRMP-2 (Thr 514) antibody. The results (Fig. 5A) show that untreated cells have readily detectable pCRMP-2 protein localized to the basal body (panels 1 – 3, arrows point to basal bodies). Importantly, all three inhibitors caused a decrease in the intensity of pCRMP-2 staining at the basal body (Lithium, panels 4 – 6; CHIR99021, panels 7 – 9; peptide inhibitor L803-mts, panels 10 – 12) in some instances below the level of detection. Quantitation of pCRMP-2 protein levels at the basal body using the protocol described in [32] shows that the reduction of pCRMP-2 at the basal body after treatment of cells with Li, CHIR99021 or the GSK-3β peptide inhibitor L803-mts was significant (P<0.05) at 60% (n = 252), 55% (n = 288) and 46% (n = 168), respectively. Levels of pCRMP-2 in the cilium were consistently below detection (Fig. 5A) suggesting that CRMP-2 in cilia is not phosphorylated.

Next, we measured the levels and localization of total CRMP-2 protein. We found that this was difficult to achieve for endogenous CRMP-2 due to the lack of a sufficiently strong specific antibody, but our results suggested that whereas ∼5% of untreated cells show CRMP-2 staining in the primary cilium, ∼30% of lithium treated cells had observable CRMP-2 staining in primary cilia to (Fig. 5B, top panels). Since this approach would not yield quantifiable data on CRMP-2 levels in the cilium after treatment with the inhibitors, we resorted to transfection of cells with full-length CRMP-2-GFP followed by treatment with GSK inhibitor. Fig. 5B shows the results, which demonstrate that upon GSK-3β inhibition by the specific CHIR99021 inhibitor, the CRMP-2 signal in the cilium was enhanced (bottom panels, arrow points to cilium) in comparison to untreated cells (middle panels, arrowhead points to basal body). Quantification of all experiments showed that CHIR99021 inhibitor caused a 50% increase in the intensity of CRMP-2-GFP in cilia (n = 48).

## Discussion

In this work we demonstrate that CRMP-2 is critically involved in primary cilium formation and that GSK-3β modulates primary cilium targeting of CRMP-2: specific inhibition of GSK-3β results in a reduction of the level of phosphorylated pCRMP-2 at the basal body and an enhanced localization of CRMP-2 at the primary cilium.

### GSK-3β and primary cilia

GSK-3β has close to 100 identified substrates [47]. Its function has been implicated in a variety of cellular processes including cell proliferation, differentiation, cell cycle control, microtubule assembly, microtubule stability, microtubule dynamics and apoptosis [48], [49]. Using lithium we recently provided evidence suggesting that GSK-3β is involved in primary cilium elongation [25] and here we confirmed this using two specific GSK-3β inhibitors. It was reported that inhibition of GSK-3β activity by indirubin-3-monoxime restores primary cilium formation in ceramide-depleted MDCK cells [50]. Downstream targets of GSK-3β remain unknown, but Von Hippel-Lindau protein - a GSK-3β target- is required for cilium function in its dephosphorylated state (after GSK-3β inactivation) [51]. In this study, we show that GSK-3β inhibition reduces CRMP-2 phosphorylation and enhances CRMP-2 localization to the primary cilium.

### A novel, GSK-3β-regulated primary cilium targeting sequence

Several studies provided evidence that specific signals are needed for targeting of proteins to the primary cilium (for a recent review see [52]). Here, we identify a new primary cilium targeting motif that differs from previously identified signals in three ways.

First, the CRMP-2 primary cilium targeting signal sequence differs from other targeting signals [52]: i) the VxPx motif in rhodopsin [39], [53], [54], PKD2 [40], the cyclic nucleotide gated channel subunit CNG1B [55] and polycystin-1 [42], ii) the AQ box (Ax^S^/_A_xQ) in somatostatin receptor 3, serotonin receptor 6, and melanocortin concentrating hormone receptor 1 [56], the ciliary localization motif identified in the KIF17 motor molecule [57], [58] and the retinitis pigmentosa 2 protein (RP2) [59] both of which require interaction with importin-β2 for trafficking to cilia. Here we found that although CRMP-2 appears to contain two VxPx motifs, mutation of these did not abolish CRMP-2 targeting to the primary cilium suggesting that they are not absolutely required for targeting of CRMP-2 to the primary cilium. We observed a 50% reduction in the level of CRMP-2 in the primary cilium, consistent with observations for CNG1B [55]. In contrast, we discovered a requirement for the presence of three GSK-3β phosphorylation sites in the CRMP-2 C-terminus: their mutation essentially blocked targeting of CRMP-2 to the primary cilium. Second, unlike targeting motifs in rhodopsin, fibrocystin and calflagin, the CRMP-2 primary cilium targeting sequence does not have motifs for myristoylation and/or palmitoylation. In the former proteins, prevention of lipidation abolishes their ciliary targeting capabilities [60]–[63]. Third, and to our knowledge uniquely, the CRMP-2 primary cilium targeting motif is not constitutively active. Efficient targeting of CRMP-2 to primary cilia is modulated by GSK-3β and requires inhibition of this protein kinase. Based on our observations, we propose a model for the targeting of CRMP-2 to the primary cilium shown in Figure 6. In this model, the phosphorylation state of CRMP-2 is regulated by GSK-3β and is crucial in the determination of whether the protein resides at the basal body (Fig. 6, phosphorylated CRMP-2) or is allowed in the primary cilium (Fig. 6, dephosphorylated CRMP-2). This model explains why the majority of CRMP-2, which is normally phosphorylated, resides at the basal body while only a small portion is in the primary cilium, even though every CRMP-2 protein harbors the primary cilium targeting sequence. Inhibition of GSK-3β results in dephosphorylation and enhanced presence in the cilium. It remains to be determined which of the three GSK-3β phosphorylation sites is critically involved in this gating process.

### CRMP-2 movement into the primary cilium

Depending on the nature of proteins, axonemal and membrane proteins may move to the primary cilia via intraflagellar transport, membrane diffusion, or both. It is not known how CRMP-2 enters into the primary cilium. Sequence analysis indicates that the CRMP-2 primary cilium targeting sequence resides within a region that was shown in neuronal cells to interact with KLC1, kinesin light chain 1 [38]. In preliminary experiments we analyzed if the 51 amino acid sequence can interact with KLC3, a kinesin light chain sperm flagellum protein we investigated which has extensive homology with KLC1 [28], [64], [65]. Our preliminary data suggest that mut7-GFP and HA-KLC3 interact (Fig. S5). This suggests the possibility that CRMP-2 may be transported into the primary cilium through association with a kinesin. This possibility is in agreement with our observations that the CRMP-2 VxPx sequences play no role, because proteins with functional VxPx motifs move into the cilium through a GTPase-mediated mechanism. Interestingly, recent results demonstrate effects of phosphorylation on kinesin-cargo dynamics, including kinesin-1/smad2, UNC51, CAMKII/KIF17 and KLC2/GluR1 [66]–[70]. Of note, the KLC2-GluR1 binding is inhibited by phosphorylation of KLC2 by GSK-3β. It is thus conceivable that the phosphorylation status of CRMP-2 GSK-3β sites regulate binding to a kinesin. This possibility awaits demonstration of CRMP-2-KLC complexes in primary cilia and the effect of phosphorylation on complex formation.

### What may be the function of CRMP-2 at the primary cilium?

In mammals, overexpression of CRMP-2 in primary hippocampal neurons or SH-SY5Y neuroblastoma cells promotes axon elongation, axon induction, and the establishment and maintenance of neuronal polarity [19], although one study in rat PC-12 cells did not support this observation [71]. We did not observe primary cilium elongation after overexpression of CRMP-2-GFP in human foreskin fibroblasts. Instead, knock down of endogenous CRMP-2 protein levels inhibited primary cilium formation. This result appears similar to one made in *C. elegans*: the *C. elegans* orthologue of human CRMP-2 is unc-33. Mutations in *unc-33* result in abnormal axon termination in many neurons [72]. A hypothetical model for CRMP-2 function could propose that it functions as a bridging protein linking the primary cilium axoneme with membrane components in an intraflagellar transport system. In support of this we observed concentrations or particles along the cilium axoneme in cells expressing wild type CRMP-2-GFP, but not in cells only expressing the 51 amino acid C-terminal CRMP-2 domain (mut7-GFP) and we show that there may be a “reservoir” of CRMP-2 at the basal body ready for transport into the primary cilium after dephosphorylation of its GSK-3β sites. This predicts that the CRMP-2 region upstream of the 51 amino acid domain associates with proteins or complexes required at the primary cilia.

In conclusion, we demonstrate that an essential neuronal protein CRMP-2, which guides neurite growth, is a critical primary cilia protein. We identified a novel regulatory mechanism for primary cilium targeting of CRMP-2 based on modulation of its phosphorylation status by GSK-3β. The identification of its GSK-3β phosphorylation sites as the cilia targeting motif informs and expands the search for ciliary proteins beyond those that have putative VxPx and other ciliary targeting motifs. Furthermore, our discovery of GSK-3β-regulated ciliary targeting together with the importance of cilia during development may open up lines of research hitherto not considered.

## Supporting Information

Figure S1 **Western blot analysis of human foreskin fibroblasts.** Cells were solubilized in SDS-sample buffer, fractionated in 12% SDS-polyacrylamide gel, blotted onto nitrocellulose, and analyzed using anti-CRMP-2 antibody. Blots were reprobed with anti-actin to control for loading and procedure. CRMP-2 is expressed as a doublet in agreement with its expression pattern in neurons. Lanes 1 and 2 represent two different isolates of fibroblast extracts.
(TIF)

Figure S2 **A scrambled siRNA does not affect the expression of CRMP-2 in transfected human foreskin fibroblasts.** Cells expressing GFP, present on the vector (green), and scrambled siRNA were analyzed by staining with anti-CRMP-2 antibodies (red). Arrows indicate centrosomes.
(TIF)

Figure S3 **Western blot analysis of mut7-GFP expressing cells.** Human foreskin fibroblasts were transfected with mut7-GFP, serum-starved and analyzed by western blotting using anti-GFP antibody. The expected doublet is seen in transfected cells.
(TIF)

Figure S4 **Inhibition of GSK-3β causes dephosphorylation of CRMP-2.** RPE cells were analyzed for expression of total CRMP-2 (panel A), phosphorylated pCRMP-2 (panel B) and actin (panel C). RPE cell extracts were analyzed by western blotting using anti-CRMP-2 antibody measuring total CRMP-2 (panel A), anti-pCRMP-2(Thr514) antibody specific for phosphorylated CRMP-2 (panel B) and anti-actin (Panel C), either from untreated (lane 1) or cells treated with lithium (lane 2). Note that phosphorylated pCRMP-2 levels are decreased after lithium treatment (panel B), which has no effect on overall CRMP-2 levels (panel A).
(TIF)

Figure S5 **The CRMP-2 primary cilium targeting sequence interacts with kinesin light chain.** Cells were co-transfected with HA-tGAP1 and mut7-GFP (lane 1; negative control) or HA-KLC3 and mut7-GFP (lane 2). Extracts were prepared from transfected cells and analyzed directly for protein expression by western blotting using a mix of anti-HA and anti-GFP antibodies (panel A) or analyzed for protein interactions by immunoprecipitation with anti-GFP antibody, followed by western blotting using anti-HA antibodies (panel B). Proteins are indicated. HA-KLC3, but not HA-tGAP1 binds mut7-GFP (panel B).
(TIF)

## References

[1] Brailov I, Bancila M, Brisorgueil MJ, Miquel MC, Hamon M, et al (2000) Localization of 5-HT(6) receptors at the plasma membrane of neuronal cilia in the rat brain. Brain Res 872: 271–275. 10924708 10.1016/s0006-8993(00)02519-1

[2] Handel M, Schulz S, Stanarius A, Schreff M, Erdtmann-Vourliotis M, et al (1999) Selective targeting of somatostatin receptor 3 to neuronal cilia. Neuroscience 89: 909–926. 10199624 10.1016/s0306-4522(98)00354-6

[3] Pazour GJ, San Agustin JT, Follit JA, Rosenbaum JL, Witman GB (2002) Polycystin-2 localizes to kidney cilia and the ciliary level is elevated in orpk mice with polycystic kidney disease. Curr Biol 12: R378–R380. 12062067 10.1016/s0960-9822(02)00877-1

[4] Rohatgi R, Milenkovic L, Scott MP (2007) Patched1 regulates hedgehog signaling at the primary cilium. Science 317: 372–376. 17641202 10.1126/science.1139740

[5] Schneider L, Clement CA, Teilmann SC, Pazour GJ, Hoffmann EK, et al (2005) PDGFRalphaalpha signaling is regulated through the primary cilium in fibroblasts. Curr Biol 15: 1861–1866. 16243034 10.1016/j.cub.2005.09.012

[6] Yoder BK, Hou X, Guay-Woodford LM (2002) The polycystic kidney disease proteins, polycystin-1, polycystin-2, polaris, and cystin, are co-localized in renal cilia. J Am Soc Nephrol 13: 2508–2516. 12239239 10.1097/01.asn.0000029587.47950.25

[7] McGlashan SR, Jensen CG, Poole CA (2006) Localization of extracellular matrix receptors on the chondrocyte primary cilium. J Histochem Cytochem 54: 1005–1014. 16651393 10.1369/jhc.5A6866.2006

[8] McGlashan SR, Cluett EC, Jensen CG, Poole CA (2008) Primary cilia in osteoarthritic chondrocytes: from chondrons to clusters. Dev Dyn 237: 2013–2020. 18330928 10.1002/dvdy.21501

[9] Goetz SC, Anderson KV (2010) The primary cilium: a signalling centre during vertebrate development. Nat Rev Genet 11: 331–344. 20395968 10.1038/nrg2774 PMC3121168

[10] Corbit KC, Aanstad P, Singla V, Norman AR, Stainier DY, et al (2005) Vertebrate Smoothened functions at the primary cilium. Nature 437: 1018–1021. 16136078 10.1038/nature04117

[11] Huangfu D, Anderson KV (2005) Cilia and Hedgehog responsiveness in the mouse. Proc Natl Acad Sci U S A 102: 11325–11330. 16061793 10.1073/pnas.0505328102 PMC1183606

[12] Nonaka S, Tanaka Y, Okada Y, Takeda S, Harada A, et al (1998) Randomization of left-right asymmetry due to loss of nodal cilia generating leftward flow of extraembryonic fluid in mice lacking KIF3B motor protein. Cell 95: 829–837. 9865700 10.1016/s0092-8674(00)81705-5

[13] Badano JL, Mitsuma N, Beales PL, Katsanis N (2006) The ciliopathies: an emerging class of human genetic disorders. Annu Rev Genomics Hum Genet 7: 125–148. 16722803 10.1146/annurev.genom.7.080505.115610

[14] Eley L, Yates LM, Goodship JA (2005) Cilia and disease. Curr Opin Genet Dev 15: 308–314. 15917207 10.1016/j.gde.2005.04.008

[15] Pazour GJ, Rosenbaum JL (2002) Intraflagellar transport and cilia-dependent diseases. Trends Cell Biol 12: 551–555. 12495842 10.1016/s0962-8924(02)02410-8

[16] Somlo S, Ehrlich B (2001) Human disease: calcium signaling in polycystic kidney disease. Curr Biol 11: R356–R360. 11369247 10.1016/s0960-9822(01)00193-2

[17] Ho PT, Tucker RW (1989) Centriole ciliation and cell cycle variability during G1 phase of BALB/c 3T3 cells. J Cell Physiol 139: 398–406. 2654143 10.1002/jcp.1041390224

[18] Rieder CL, Jensen CG, Jensen LC (1979) The resorption of primary cilia during mitosis in a vertebrate (PtK1) cell line. J Ultrastruct Res 68: 173–185. 480410 10.1016/s0022-5320(79)90152-7

[19] Inagaki N, Chihara K, Arimura N, Menager C, Kawano Y, et al (2001) CRMP-2 induces axons in cultured hippocampal neurons. Nat Neurosci 4: 781–782. 11477421 10.1038/90476

[20] Cole AR, Knebel A, Morrice NA, Robertson LA, Irving AJ, et al (2004) GSK-3 phosphorylation of the Alzheimer epitope within collapsin response mediator proteins regulates axon elongation in primary neurons. J Biol Chem 279: 50176–50180. 15466863 10.1074/jbc.C400412200 PMC1832086

[21] Yoshimura T, Kawano Y, Arimura N, Kawabata S, Kikuchi A, et al (2005) GSK-3beta regulates phosphorylation of CRMP-2 and neuronal polarity. Cell 120: 137–149. 15652488 10.1016/j.cell.2004.11.012

[22] Minturn JE, Fryer HJ, Geschwind DH, Hockfield S (1995) TOAD-64, a gene expressed early in neuronal differentiation in the rat, is related to unc-33, a C. elegans gene involved in axon outgrowth. J Neurosci 15: 6757–6766. 7472434 10.1523/JNEUROSCI.15-10-06757.1995 PMC6578000

[23] Ricard D, Stankoff B, Bagnard D, Aguera M, Rogemond V, et al (2000) Differential expression of collapsin response mediator proteins (CRMP/ULIP) in subsets of oligodendrocytes in the postnatal rodent brain. Mol Cell Neurosci 16: 324–337. 11085871 10.1006/mcne.2000.0888

[24] Rahajeng J, Giridharan SS, Naslavsky N, Caplan S (2010) Collapsin response mediator protein-2 (Crmp2) regulates trafficking by linking endocytic regulatory proteins to dynein motors. J Biol Chem 285: 31918–31922. 20801876 10.1074/jbc.C110.166066 PMC2952192

[25] Ou Y, Ruan Y, Cheng M, Moser JJ, Rattner JB, et al (2009) Adenylate cyclase regulates elongation of mammalian primary cilia. Exp Cell Res 315: 2802–2817. 19576885 10.1016/j.yexcr.2009.06.028 PMC3161028

[26] Kaidanovich-Beilin O, Milman A, Weizman A, Pick CG, Eldar-Finkelman H (2004) Rapid antidepressive-like activity of specific glycogen synthase kinase-3 inhibitor and its effect on beta-catenin in mouse hippocampus. Biol Psychiatry 55: 781–784. 15050857 10.1016/j.biopsych.2004.01.008

[27] Plotkin B, Kaidanovich O, Talior I, Eldar-Finkelman H (2003) Insulin mimetic action of synthetic phosphorylated peptide inhibitors of glycogen synthase kinase-3. J Pharmacol Exp Ther 305: 974–980. 12626660 10.1124/jpet.102.047381

[28] Zhang Y, Oko R, van der Hoorn FA (2004) Rat kinesin light chain 3 associates with spermatid mitochondria. Dev Biol 275: 23–33. 15464570 10.1016/j.ydbio.2004.07.014 PMC3138780

[29] Fitzgerald C, Sikora C, Lawson V, Dong K, Cheng M, et al (2006) Mammalian transcription in support of hybrid mRNA and protein synthesis in testis and lung. J Biol Chem 281: 38172–38180. 17040916 10.1074/jbc.M606010200 PMC3158134

[30] Ou Y, Rattner JB (2000) A subset of centrosomal proteins are arranged in a tubular conformation that is reproduced during centrosome duplication. Cell Motil Cytoskeleton 47: 13–24. 11002307 10.1002/1097-0169(200009)47:1<13::AID-CM2>3.0.CO;2-C

[31] Ou YY, Mack GJ, Zhang M, Rattner JB (2002) CEP110 and ninein are located in a specific domain of the centrosome associated with centrosome maturation. J Cell Sci 115: 1825–1835. 11956314 10.1242/jcs.115.9.1825

[32] Mahjoub MR, Stearns T (2012) Supernumerary Centrosomes Nucleate Extra Cilia and Compromise Primary Cilium Signaling. Curr Biol . In press. S0960-9822(12)00733-6 [pii];10.1016/j.cub.2012.06.057 [doi]. 10.1016/j.cub.2012.06.057 PMC4094149 22840514

[33] Rattner JB, Sciore P, Ou Y, van der Hoorn FA, Lo IK (2010) Primary cilia in fibroblast-like type B synoviocytes lie within a cilium pit: a site of endocytosis. Histol Histopathol 25: 865–875. 20503175 10.14670/HH-25.865

[34] Gu Y, Hamajima N, Ihara Y (2000) Neurofibrillary tangle-associated collapsin response mediator protein-2 (CRMP-2) is highly phosphorylated on Thr-509, Ser-518, and Ser-522. Biochemistry 39: 4267–4275. 10757975 10.1021/bi992323h

[35] Fukata Y, Itoh TJ, Kimura T, Menager C, Nishimura T, et al (2002) CRMP-2 binds to tubulin heterodimers to promote microtubule assembly. Nat Cell Biol 4: 583–591. 12134159 10.1038/ncb825

[36] Arimura N, Hattori A, Kimura T, Nakamuta S, Funahashi Y, et al (2009) CRMP-2 directly binds to cytoplasmic dynein and interferes with its activity. J Neurochem 111: 380–390. 19659462 10.1111/j.1471-4159.2009.06317.x

[37] Nishimura T, Fukata Y, Kato K, Yamaguchi T, Matsuura Y, et al (2003) CRMP-2 regulates polarized Numb-mediated endocytosis for axon growth. Nat Cell Biol 5: 819–826. 12942088 10.1038/ncb1039

[38] Kimura T, Watanabe H, Iwamatsu A, Kaibuchi K (2005) Tubulin and CRMP-2 complex is transported via Kinesin-1. J Neurochem 93: 1371–1382. 15935053 10.1111/j.1471-4159.2005.03063.x

[39] Deretic D, Schmerl S, Hargrave PA, Arendt A, McDowell JH (1998) Regulation of sorting and post-Golgi trafficking of rhodopsin by its C-terminal sequence QVS(A)PA. Proc Natl Acad Sci U S A 95: 10620–10625. 9724753 10.1073/pnas.95.18.10620 PMC27944

[40] Geng L, Okuhara D, Yu Z, Tian X, Cai Y, et al (2006) Polycystin-2 traffics to cilia independently of polycystin-1 by using an N-terminal RVxP motif. J Cell Sci 119: 1383–1395. 16537653 10.1242/jcs.02818

[41] Mazelova J, Astuto-Gribble L, Inoue H, Tam BM, Schonteich E, et al (2009) Ciliary targeting motif VxPx directs assembly of a trafficking module through Arf4. EMBO J 28: 183–192. 19153612 10.1038/emboj.2008.267 PMC2637330

[42] Ward HH, Brown-Glaberman U, Wang J, Morita Y, Alper SL, et al (2011) A conserved signal and GTPase complex are required for the ciliary transport of polycystin-1. Mol Biol Cell 22: 3289–3305. 21775626 10.1091/mbc.E11-01-0082 PMC3172256

[43] Uchida Y, Ohshima T, Sasaki Y, Suzuki H, Yanai S, et al (2005) Semaphorin3A signalling is mediated via sequential Cdk5 and GSK3beta phosphorylation of CRMP2: implication of common phosphorylating mechanism underlying axon guidance and Alzheimer's disease. Genes Cells 10: 165–179. 15676027 10.1111/j.1365-2443.2005.00827.x

[44] Cole AR, Causeret F, Yadirgi G, Hastie CJ, McLauchlan H, et al (2006) Distinct priming kinases contribute to differential regulation of collapsin response mediator proteins by glycogen synthase kinase-3 in vivo. J Biol Chem 281: 16591–16598. 16611631 10.1074/jbc.M513344200 PMC1805471

[45] Yang J, Takahashi Y, Cheng E, Liu J, Terranova PF, et al (2010) GSK-3beta promotes cell survival by modulating Bif-1-dependent autophagy and cell death. J Cell Sci 123: 861–870. 20159967 10.1242/jcs.060475 PMC2831760

[46] Rao R, Hao CM, Redha R, Wasserman DH, McGuinness OP, et al (2007) Glycogen synthase kinase 3 inhibition improves insulin-stimulated glucose metabolism but not hypertension in high-fat-fed C57BL/6J mice. Diabetologia 50: 452–460. 17151860 10.1007/s00125-006-0552-5

[47] Sutherland C (2011) What Are the bona fide GSK3 Substrates? Int J Alzheimers Dis 2011: 505607. 21629754 10.4061/2011/505607 PMC3100594

[48] Zhou FQ, Snider WD (2005) CELL BIOLOGY: GSK-3beta and Microtubule Assembly in Axons. Science 308: 211–214. 15825222 10.1126/science.1110301

[49] Xu C, Kim NG, Gumbiner BM (2009) Regulation of protein stability by GSK3 mediated phosphorylation. Cell Cycle 8: 4032–4039. 19923896 10.4161/cc.8.24.10111 PMC2824240

[50] Wang G, Krishnamurthy K, Bieberich E (2009) Regulation of primary cilia formation by ceramide. J Lipid Res 50: 2103–2110. 19372594 10.1194/jlr.M900097-JLR200 PMC2739757

[51] Thoma CR, Frew IJ, Hoerner CR, Montani M, Moch H, et al (2007) pVHL and GSK3beta are components of a primary cilium-maintenance signalling network. Nat Cell Biol 9: 588–595. 17450132 10.1038/ncb1579

[52] Nachury MV, Seeley ES, Jin H (2010) Trafficking to the ciliary membrane: how to get across the periciliary diffusion barrier? Annu Rev Cell Dev Biol 26 59–87 59–87. 10.1146/annurev.cellbio.042308.113337 PMC2952038 19575670

[53] Sung CH, Makino C, Baylor D, Nathans J (1994) A rhodopsin gene mutation responsible for autosomal dominant retinitis pigmentosa results in a protein that is defective in localization to the photoreceptor outer segment. J Neurosci 14: 5818–5833. 7523628 10.1523/JNEUROSCI.14-10-05818.1994 PMC6576989

[54] Li T, Snyder WK, Olsson JE, Dryja TP (1996) Transgenic mice carrying the dominant rhodopsin mutation P347S: evidence for defective vectorial transport of rhodopsin to the outer segments. Proc Natl Acad Sci U S A 93: 14176–14181. 8943080 10.1073/pnas.93.24.14176 PMC19513

[55] Jenkins PM, Hurd TW, Zhang L, McEwen DP, Brown RL, et al (2006) Ciliary targeting of olfactory CNG channels requires the CNGB1b subunit and the kinesin-2 motor protein, KIF17. Curr Biol 16: 1211–1216. 16782012 10.1016/j.cub.2006.04.034

[56] Berbari NF, Johnson AD, Lewis JS, Askwith CC, Mykytyn K (2008) Identification of ciliary localization sequences within the third intracellular loop of G protein-coupled receptors. Mol Biol Cell 19: 1540–1547. 18256283 10.1091/mbc.E07-09-0942 PMC2291422

[57] Verhey KJ, Dishinger J, Kee HL (2011) Kinesin motors and primary cilia. Biochem Soc Trans 39: 1120–1125. 21936775 10.1042/BST0391120 PMC3538878

[58] Dishinger JF, Kee HL, Jenkins PM, Fan S, Hurd TW, et al (2010) Ciliary entry of the kinesin-2 motor KIF17 is regulated by importin-beta2 and RanGTP. Nat Cell Biol 12: 703–710. 20526328 10.1038/ncb2073 PMC2896429

[59] Hurd TW, Fan S, Margolis BL (2011) Localization of retinitis pigmentosa 2 to cilia is regulated by Importin beta2. J Cell Sci 124: 718–726. 21285245 10.1242/jcs.070839 PMC3039017

[60] Godsel LM, Engman DM (1999) Flagellar protein localization mediated by a calcium-myristoyl/palmitoyl switch mechanism. EMBO J 18: 2057–2065. 10205160 10.1093/emboj/18.8.2057 PMC1171290

[61] Tao B, Bu S, Yang Z, Siroky B, Kappes JC, et al (2009) Cystin localizes to primary cilia via membrane microdomains and a targeting motif. J Am Soc Nephrol 20: 2570–2580. 19850956 10.1681/ASN.2009020188 PMC2794227

[62] Follit JA, Li L, Vucica Y, Pazour GJ (2010) The cytoplasmic tail of fibrocystin contains a ciliary targeting sequence. J Cell Biol 188: 21–28. 20048263 10.1083/jcb.200910096 PMC2812845

[63] Tam BM, Moritz OL, Hurd LB, Papermaster DS (2000) Identification of an outer segment targeting signal in the COOH terminus of rhodopsin using transgenic Xenopus laevis. J Cell Biol 151: 1369–1380. 11134067 10.1083/jcb.151.7.1369 PMC2150681

[64] Junco A, Bhullar B, Tarnasky HA, van der Hoorn FA (2001) Kinesin light-chain KLC3 expression in testis is restricted to spermatids. Biol Reprod 64: 1320–1330. 11319135 10.1095/biolreprod64.5.1320 PMC3161965

[65] Bhullar B, Zhang Y, Junco A, Oko R, van der Hoorn FA (2003) Association of Kinesin light chain with Outer dense fibers in a Microtubule-independent fashion. J Biol Chem 278: 16159–16168. 12594206 10.1074/jbc.M213126200 PMC3178653

[66] Manser C, Guillot F, Vagnoni A, Davies J, Lau KF, et al.. (2011) Lemur tyrosine kinase-2 signalling regulates kinesin-1 light chain-2 phosphorylation and binding of Smad2 cargo. Oncogene 10. 10.1038/onc.2011.437 PMC3272475 21996745

[67] Toda H, Mochizuki H, Flores R III, Josowitz R, Krasieva TB, et al (2008) UNC-51/ATG1 kinase regulates axonal transport by mediating motor-cargo assembly. Genes Dev 22: 3292–3307. 19056884 10.1101/gad.1734608 PMC2600757

[68] Ally S, Jolly AL, Gelfand VI (2008) Motor-cargo release: CaMKII as a traffic cop. Nat Cell Biol 10: 3–5. 18172423 10.1038/ncb0108-3

[69] Guillaud L, Wong R, Hirokawa N (2008) Disruption of KIF17-Mint1 interaction by CaMKII-dependent phosphorylation: a molecular model of kinesin-cargo release. Nat Cell Biol 10: 19–29. 18066053 10.1038/ncb1665

[70] Du J, Wei Y, Liu L, Wang Y, Khairova R, et al (2010) A kinesin signaling complex mediates the ability of GSK-3beta to affect mood-associated behaviors. Proc Natl Acad Sci U S A 107: 11573–11578. 20534517 10.1073/pnas.0913138107 PMC2895136

[71] Bork K, Karbe Y, Pollscheit J, Glaubitz N, Nohring S, et al (2010) Role of collapsin response mediator protein-2 in neurite outgrowth of PC12 cells. Neuroreport 21: 641–645. 20453694 10.1097/WNR.0b013e32833a7d53

[72] Hedgecock EM, Culotti JG, Thomson JN, Perkins LA (1985) Axonal guidance mutants of Caenorhabditis elegans identified by filling sensory neurons with fluorescein dyes. Dev Biol 111: 158–170. 3928418 10.1016/0012-1606(85)90443-9

