# Supplementary figures and images for: Targeting of CRMP-2 to the Primary Cilium Is Modulated by GSK-3β
Source: PLoS One. 2012 Nov 21;7(11):e48773. doi: 10.1371/journal.pone.0048773 (PMC3504062; doi:10.1371/journal.pone.0048773)

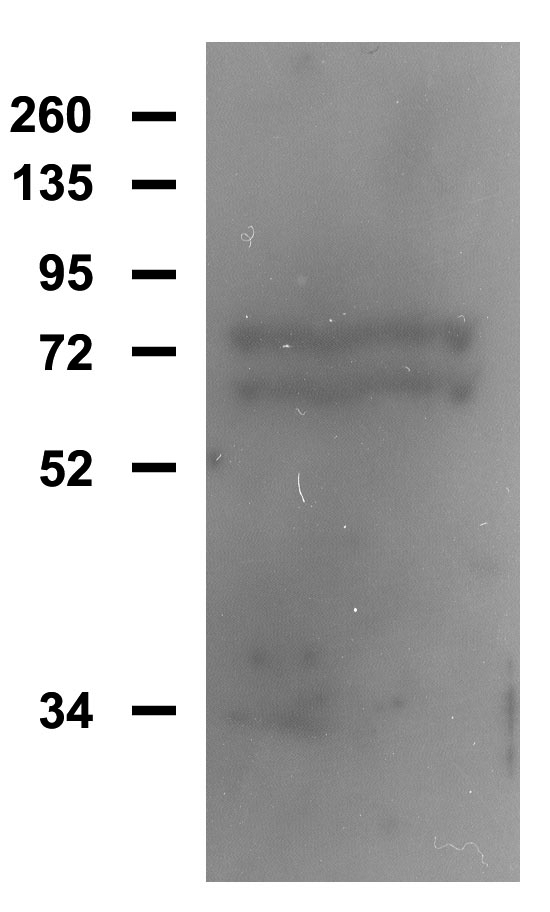

Supplement: Figure S1 — Western blot analysis of human foreskin fibroblasts. Cells were solubilized in SDS-sample buffer, fractionated in 12% SDS-polyacrylamide gel, blotted onto nitrocellulose, and analyzed using anti-CRMP-2 antibody. Blots were reprobed with anti-actin to control for loading and procedure. CRMP-2 is expressed as a doublet in agreement with its expression pattern in neurons. Lanes 1 and 2 represent two different isolates of fibroblast extracts. (TIF) [file pone.0048773.s001.tif]

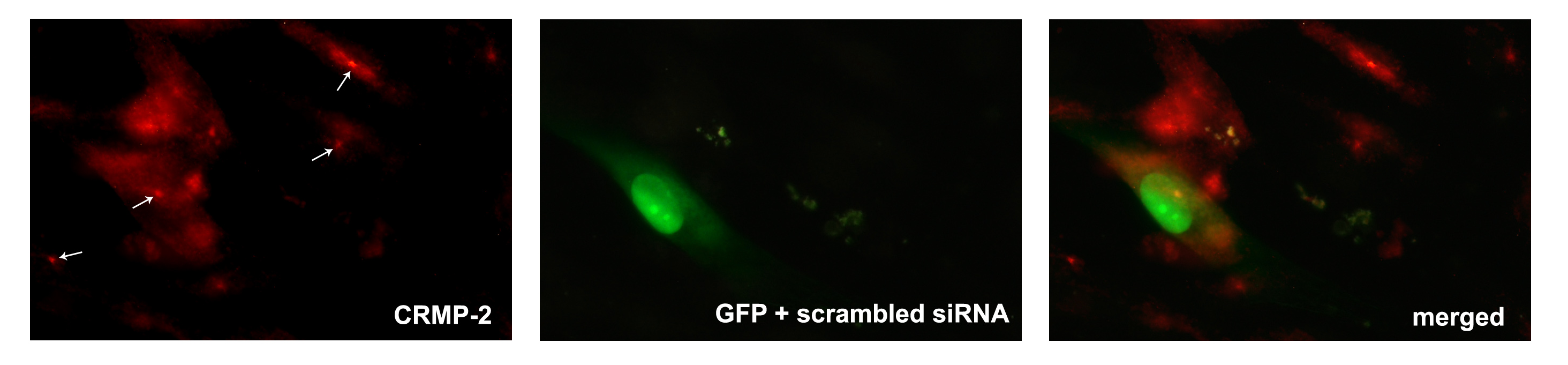

Supplement: Figure S2 — A scrambled siRNA does not affect the expression of CRMP-2 in transfected human foreskin fibroblasts. Cells expressing GFP, present on the vector (green), and scrambled siRNA were analyzed by staining with anti-CRMP-2 antibodies (red). Arrows indicate centrosomes. (TIF) [file pone.0048773.s002.tif]

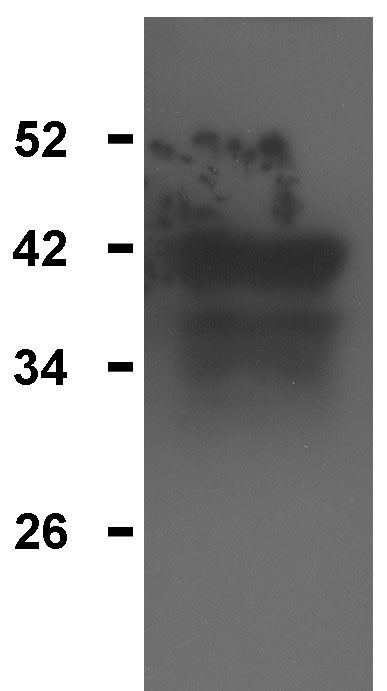

Supplement: Figure S3 — Western blot analysis of mut7-GFP expressing cells. Human foreskin fibroblasts were transfected with mut7-GFP, serum-starved and analyzed by western blotting using anti-GFP antibody. The expected doublet is seen in transfected cells. (TIF) [file pone.0048773.s003.tif]

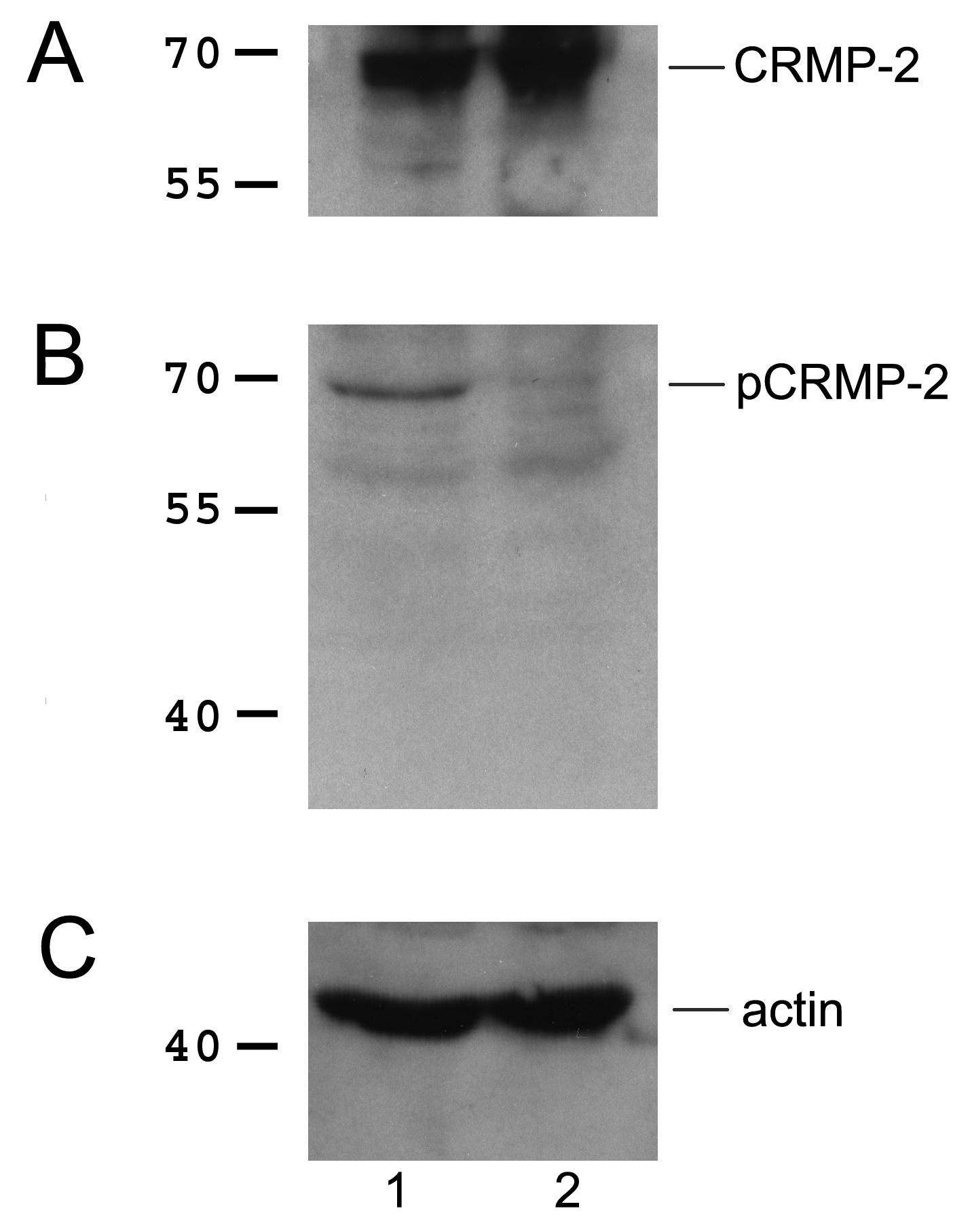

Supplement: Figure S4 — Inhibition of GSK-3β causes dephosphorylation of CRMP-2. RPE cells were analyzed for expression of total CRMP-2 (panel A), phosphorylated pCRMP-2 (panel B) and actin (panel C). RPE cell extracts were analyzed by western blotting using anti-CRMP-2 antibody measuring total CRMP-2 (panel A), anti-pCRMP-2(Thr514) antibody specific for phosphorylated CRMP-2 (panel B) and anti-actin (Panel C), either from untreated (lane 1) or cells treated with lithium (lane 2). Note that phosphorylated pCRMP-2 levels are decreased after lithium treatment (panel B), which has no effect on overall CRMP-2 levels (panel A). (TIF) [file pone.0048773.s004.tif]

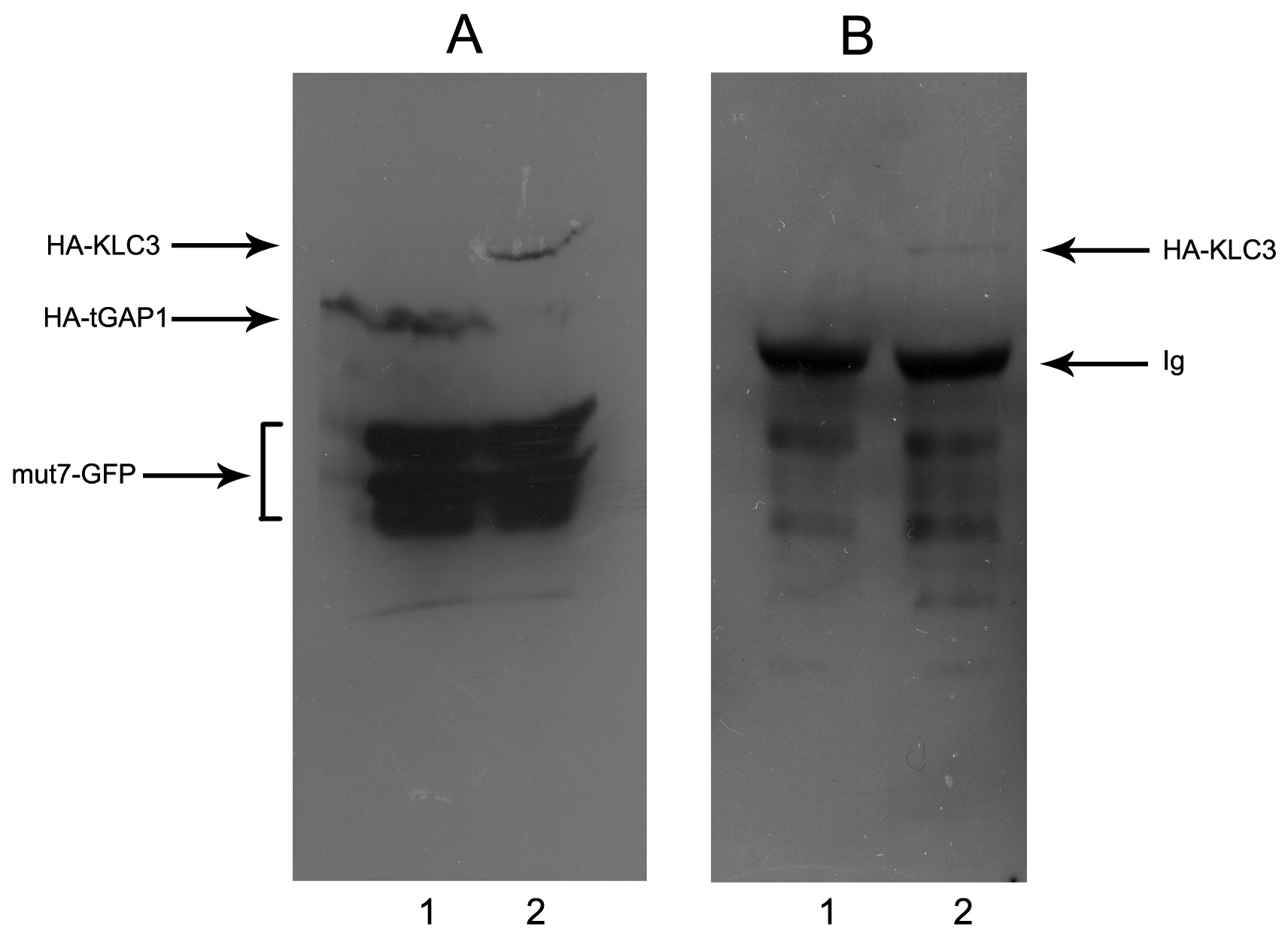

Supplement: Figure S5 — The CRMP-2 primary cilium targeting sequence interacts with kinesin light chain. Cells were co-transfected with HA-tGAP1 and mut7-GFP (lane 1; negative control) or HA-KLC3 and mut7-GFP (lane 2). Extracts were prepared from transfected cells and analyzed directly for protein expression by western blotting using a mix of anti-HA and anti-GFP antibodies (panel A) or analyzed for protein interactions by immunoprecipitation with anti-GFP antibody, followed by western blotting using anti-HA antibodies (panel B). Proteins are indicated. HA-KLC3, but not HA-tGAP1 binds mut7-GFP (panel B). (TIF) [file pone.0048773.s005.tif]
